# Sharing electronically and accessibly in library-led instruction

**DOI:** 10.5195/jmla.2021.1361

**Published:** 2021-10-01

**Authors:** Julia Jankovic Dahm, Julia Grace Reese

**Affiliations:** 1 jdahm@pitt.edu, Assistant Director for Technology Integration and Fulfillment Services, Health Sciences Library System, University of Pittsburgh, Pittsburgh, PA; 2 jgr12@pitt.edu, Technology Integration Services Administrator, Health Sciences Library System, University of Pittsburgh, Pittsburgh, PA

## Abstract

The electronic information and technology accessibility project is a strategic overhauling of the digital instructional materials of the Health Sciences Library System (HSLS) to comply with the accessibility standards established in a 2020 University of Pittsburgh policy. Though these technologies have existed for quite some time, library instructors were not skilled in the actual creation and design of documents, web content, and presentations with accessibility in mind. Over the past year and a half, a team within HSLS developed detailed guidance and education on universal design and creating an inclusive online learning environment. These guidelines were developed in accordance with Section 508 and the WCAG2.1, with a focus on an improved experience for the D/deaf community and those with visual impairments. We initially made accessibility improvements to online subject guides, in-person presentations, and digitally shared class materials. The COVID-19 pandemic and complete shift to virtual instruction then necessitated the evaluation of platforms used in remote learning (such as Zoom and Panopto), where accessibility best practices needed to be incorporated. This article highlights going beyond in-program accessibility checkers and describes how library technology experts and content creators worked together to bridge the gap of accessibility in the information we share.

Digital instructional materials that are formatted for accessibility are essential to ensuring equivalent access to information, especially for those using assistive technology. The University of Pittsburgh's planning and implementation of the Electronic Information and Technology Accessibility Policy in March 2020 prompted the Health Sciences Library System (HSLS) to assess and improve the accessibility of the library's digital instructional content [1]. By also incorporating the principles of universal design into accessibility efforts, a more inclusive learning environment was created for all end users.

All HSLS accessibility initiatives were based on WCAG2.1 and Section 508 guidelines. WCAG2.1 is a set of recommendations developed by individuals and organizations internationally to provide a shared standard of accessibility [2]. Section508.gov reflects requirements of Section 508 of the Rehabilitation Act and provides guidance for US federal agency staff with a role in IT accessibility [3]. Both WCAG2.1 and Section 508 are widely accepted and used nationally and internationally as accessibility standards.

HSLS's primary goal was to construct a workflow that would enable library instructors to proactively create accessible content. HSLS aimed to surpass improvements recommended by accessibility checkers available in Microsoft Office and Adobe Acrobat, as these do not comprehensively identify compliance issues. Therefore, step-by-step instructional guides and checklists for authoring accessible Microsoft Word, PowerPoint, Excel, and Adobe PDF files were developed (Table 1). PowerPoint template files with accessible font sizes and color use were also created for instructor presentations.

**Table 1:** Content from four step-by-step guides that provided instructions on fixing accessibility issues in documents and files (table is not comprehensive)

Areas to address accessibility	Accessibility considerations
Text formatting	Readable font size; sans serif font used; adequate spacing between lines of text
Use of color, images, figures, tables, and audio/video	Adequate color contrast used in text and figures; descriptive alt text available for images, figures, and tables; closed captioning available for audio/video
Document structure and reading order	Heading styles used appropriately; reading order set in PPTX and PDF files
File properties and security settings	Document title and language identified; security settings disabled
Digital file sharing practices	Descriptive file name used; DOCX, PPTX, and XLSX files shared instead of PDF version when possible

As a commonly used instructional tool, HSLS LibGuides were included in initial efforts. Principles of universal design were utilized to create a new site template, which featured a side-based navigation menu, single-column format, and standardized style. Step-by-step instructions and a checklist for creating accessible web content were also developed for LibGuide authors (Table 2). Browser extensions that evaluate site accessibility (Siteimprove, WAVE, and tota11y) were used to help identify accessibility issues in individual LibGuides.

**Table 2:** Content from a step-by-step guide that provided instructions on fixing accessibility issues in web content, specific to the LibGuide editing platform (table is not comprehensive)

Areas to address accessibility	Accessibility considerations
Use of graphics, audio/video, and tables	Descriptive alt text available for graphics; closed captioning available for audio/video; table header(s) identified; table summary and caption available
Hyperlink formatting	Hyperlinked text accurately describes link destination; hyperlinked text does not depend on surrounding content to achieve meaning
Headings	Built-in heading styles used instead of manually creating the look of headings; heading styles used in correct hierarchy
Non-HTML content	Linked documents and files are fully accessible; fillable forms are accessibly formatted

A key to success was identifying a staff member in Digital Library Services to manage the project and become well versed in digital accessibility. This staff member created the step-by-step accessibility guides described above and hosted information sessions for library instructors throughout the project. In May 2020, library instructors attended an introductory hour-long session to learn about basic accessibility principles and their responsibilities in providing accessible instruction. In July 2020, a recorded video was shared demonstrating the more complicated steps of creating accessible PowerPoints, which had been identified as a pain point for many instructors. In January 2021, a final hour-long session was held to outline recent updates to the library's accessibility policies and reiterate instructors' main responsibilities. At this session, instructors were also invited to voice any problems or concerns they had with the policies and their implementation.

At the above sessions, timelines for bringing materials into compliance were communicated to instructors. Newly created instructional materials were prioritized with a deadline to be made accessible by September 2020. In January 2021, instructors were asked to update materials they had created prior to the adoption of the policy. Staggering these deadlines eased instructor workload and paralleled the priorities established by the university-wide policy.

HSLS's accessibility project coincided with the switch to all-virtual instruction caused by the COVID-19 pandemic. To ensure the inclusivity of the newly remote learning environment, accessibility reviews were conducted for Zoom (used for live-teaching sessions) [4] and Panopto (used to host recorded instructional videos) [5]. In January 2021, guides describing the accessibility of these platforms' features and suggested accommodations were distributed to instructors. Video captioning was implemented partially in summer 2020 through the use of autogenerated captions; student workers were then hired in January 2021 to review and correct errors in autogenerated captions.

Although vetted guidelines were used to develop HSLS's accessibility polices, HSLS did not test the accessibility of its instructional materials with users who readily use screen readers or with those who rely on captions for understanding audio. There are ongoing concerns regarding the oversight and compliance with accessibility, because guidelines must be followed each time library instructors create or change materials. However, yearly reporting within the University and occasional internal screening of materials and classes should help maintain overall compliance with accessibility best practices.

As HSLS transitions back to an in-person instructional environment, the Digital Library Services team plans to expand the accessibility review beyond digital instructional materials to include in-person instructional materials, software used in instruction, and classroom accessibility. This work will build upon the library's existing accommodation policy that is shared with all class registrants.
